# Plasma levels of autophagy regulator Rubicon are inversely associated with acute coronary syndrome

**DOI:** 10.3389/fcvm.2023.1279899

**Published:** 2024-01-05

**Authors:** Marie-Hélène Grazide, Jean-Bernard Ruidavets, Wim Martinet, Meyer Elbaz, Cécile Vindis

**Affiliations:** ^1^ Center for Clinical Investigation (CIC1436)/CARDIOMET, Rangueil University Hospital, Toulouse, France; ^2^ University of Toulouse III, Toulouse, France; ^3^ Department of Epidemiology, INSERM UMR 1027, Toulouse, France; ^4^ Laboratory of Physiopharmacology, University of Antwerp, Antwerp, Belgium; ^5^ Department of Cardiology, Rangueil University Hospital, Toulouse, France

**Keywords:** Rubicon, autophagy regulator, biomarker, acute coronary syndrome, risk prediction

## Abstract

**Background:**

The discovery of novel biomarkers that improve current cardiovascular risk prediction models of acute coronary syndrome (ACS) is needed for the identification of very high-risk patients and therapeutic decision-making. Autophagy is a highly conserved catabolic mechanism for intracellular degradation of cellular components through lysosomes. The autophagy process helps maintain cardiac homeostasis and dysregulated autophagy has been described in cardiovascular conditions. Rubicon (Run domain Beclin-1-interacting and cysteine-rich domain-containing protein) is a key regulator of autophagy with a potential role in cardiac stress.

**Objectives:**

The aims of the present study were to assess whether changes in circulating Rubicon levels are associated with ACS and to evaluate the added value of Rubicon to a clinical predictive risk model.

**Methods and results:**

The study population included ACS patients (*n* = 100) and control subjects (*n* = 99) at high to very high cardiovascular risk but without known coronary event. Plasma Rubicon levels were measured in the whole study population by enzyme-linked immunosorbent assay. Multivariate logistic regression analyses established that Rubicon levels were inversely associated with ACS. A receiver operating characteristic curve analysis demonstrated that the addition of Rubicon improved the predictive performance of the model with an increased area under the curve from 0.868 to 0.896 (*p* = 0.038).

**Conclusions:**

Plasma levels of the autophagy regulator Rubicon are associated with ACS and provide added value to classical risk markers for ACS.

## 1 Introduction

Cardiovascular disease (CVD) ranks as one of the most prevalent causes of morbidity and mortality worldwide. Despite considerable progresses in acute care and secondary prevention after acute coronary syndrome (ACS), the global burden is expected to rise because of the aging of the world population and increased obesity and diabetes (1). In this scenario, there is still a need to discover novel biomarkers for the prognostication and therapeutic decision-making. Autophagy is a highly conserved cellular catabolic process that is responsible for the destruction of long-lived proteins and organelles via a lysosome-dependent pathway. In the general form of autophagy, cytoplasmic cargo targeted for destruction is sequestered inside double-membrane vesicles called autophagosomes and is delivered to the lysosome by fusion for breakdown (2). This process is crucial for preserving cellular homeostasis and dysregulated autophagy has been associated to the pathogenesis of a wide array of diseases including cardiovascular conditions such as myocardial ischemia-reperfusion (I/R) (3–5). Of note, myocardial I/R is associated with type 1 myocardial infarction (MI) which is the consequence of atherosclerotic plaque rupture. The categories of patients with type 1 ST-elevation MI (STEMI) or type 1 non-ST-elevation MI (NSTEMI) are customarily included in the definition of ACS (6).

Once activated, autophagy proceeds through four sequential steps, each step requiring specific regulatory proteins and complexes that have been well described in several reviews and will not be detailed here (7). Ischemic injury activates autophagy in cardiomyocytes enabling them to cope with nutritional stress and improve cell survival (8–10). Nah and colleagues showed that autosis, a novel form of cell death caused by excessive autophagy, occurred during the late reperfusion phase in the mouse heart exposed to I/R. Notably, autosis is associated with reduced autophagic flux, blockade of autophagosome clearance and upregulation of Rubicon (RUN domain Beclin-1 interacting and cysteine-rich-containing protein) (11). Rubicon is one of the few endogenous inhibitors of autophagy during autophagosome maturation and autophagosome-lysosome fusion (12, 13). Rubicon has been also involved in autophagy-independent functions including LAP (LC3-associated phagocytosis), endosomal trafficking and inflammatory responses (14–16). The close relationship between Rubicon and myocardial I/R (11, 17) makes it as a potential biomarker for measuring autophagy level in clinical practice and a therapeutic target for modulating autophagy. At present, there is a lack of studies on the levels of circulating autophagy proteins as easily accessible diagnostic or prognostic tools for monitoring cardiac diseases even though autophagy is known to play a major role. Thanks to commercially available immunoassay kits, a panel of autophagy proteins were measured in human body fluids. Recent work has demonstrated that circulating levels of the autophagy/mitophagy proteins ATG5 and Parkin were found as diagnostic tools for early monitoring of patients with cognitive decline, for identifying the active phase of multiple sclerosis or related to the severity of hypoxic injury during perinatal hypoxic-ischemic encephalopathy (18–20). In addition, a correlation has been established between the level of serum circulating Beclin1 and the degree of airflow obstruction in COPD patients compared to healthy controls (21). Interestingly, Emmanuele and colleagues showed that healthy centenarians have increased circulating Beclin1 protein levels in comparison to a population of young healthy subjects or patients with MI (22). Thus, for our work we sought for the presence of Rubicon in the circulation and its possible association with myocardial infarction. At this end, using immunoassays we measured plasma Rubicon protein levels in a case-control study including patients who experienced ACS and very high-risk asymptomatic individuals according to a SCORE (Systematic Coronary Risk Evaluation) model (23).

## 2 Materials and methods

### 2.1 Study population

The population is a case-control study including ACS patients from a prospective cohort (NCT02405468) (24). Cases (*n* = 100, men and women) were patients who experienced an acute ischemic event and were enrolled 3 days after the acute ischemic phase (henceforward referred to as ACS patients). Demographic, clinical, and biological variables were recorded and a blood sample was collected. Coronary data and left ventricular ejection fraction were accessible for all patients. During the same period, subjects (*n* = 99, men and women) at very high cardiovascular risk (henceforward referred to as controls) but without any previous vascular or coronary event, were recruited in the Center for the Prevention of Cardiovascular Disease in the Cardiology Department (University Hospital Center, Toulouse, France). The exclusion criteria for the both populations included: infectious disease within 1 week before enrollment, immunocompromised patients, antibiotic treatment within 1 month before enrollment, chronic viral infection, chronic inflammatory intestinal bowel disease, renal failure (estimated glomerular filtration rate <50 ml/min per 1.73 m^2^) and pregnancy. The written informed consent was obtained from each individual. The study protocol conformed to the ethical guidelines of the 1975 Declaration of Helsinki and Toulouse Hospital guidelines. The study protocol was previously reviewed and approved by the regional board of health authorities (Agence Régionale de Santé Occitanie-Midi-Pyrénées, Toulouse, France) and the Institution's ethics committee on research on humans (Comité de protection des personnes du Sud-Ouest, Toulouse, France).

### 2.2 Blood sampling and plasma levels of Rubicon determination

EDTA plasma samples were prepared and stored at −80°C within a 3 h' delay. Rubicon were measured by using commercially available enzyme-linked immunosorbent assay kits [human Rubicon ELISA Kit (HUFI06681), Assay Genie, Dublin, Ireland]. Briefly, the ELISA Rubicon assay is a quantitative sandwich ELISA technique and was done on frozen (−80°C) EDTA plasma samples never thawed (haemolyzed samples were excluded). Once the samples (100 µl in each well), blanks and standards were plated, the plate is incubated with the specific biotin-conjugated primary antibody Rubicon and Avidin conjugated Horseradish Peroxidase. The signal intensity (yellow color) is measured on microplate reader at 450 nm.

### 2.3 Statistical analysis

Data are presented as means and standard deviations for quantitative variables and percentages for categorical variables. The distribution of qualitative variables between cases and controls was compared using the Chi2 test. Fisher's exact test was used when basic assumptions were not satisfied. The student's *t*-test was used to compare mean values of quantitative variables. The Shapiro–Wilk's and Levene's tests were applied to assess the normality of distribution of residuals and the homogeneity of variances. A logarithmic transformation of the variables or a Wilcoxon–Mann–Whitney test was achieved when basic assumptions of Student's *t*-test were not satisfied. The autophagy marker Rubicon was compared between cases and controls using the Mann and Whitney test. Relationships between the levels of Rubicon and metabolic parameters or cardiovascular risk factors were tested with a non-parametric measure of rank correlation (Spearman rank correlation). Autophagy factor was examined in a multivariate analysis by adjusting for variables of the basic model (gender, current smoker, hypertension, dyslipidemia, diabetes, obesity, age and heredity). Medical treatments were added in the basic model. Logistic regression analyses were performed with polynomial models (quadratic and cubic) to examine for possible non-linear relationships between continuous variables and case-control status. Because of a clearly non-linear relationship with Rubicon, logistic regression analyses were carried out using a categorization of the variable into quartiles. ROC curve analysis was performed to establish the capacity to discriminate cases and controls for Rubicon, the calculation of the standard error of the area under the curve (AUC) was done applying the Delong's method. All tests were two-tailed at the level of significance of 0.05. Analyses were achieved using SAS software, version 9.4 (SAS Institute, Cary, NC, USA) and STATA statistical software, release 14.1 (Stata Corporation, College Station, TX, USA).

## 3 Results

### 3.1 Population characteristics and analysis of Rubicon plasma concentrations

**Figure 1:**
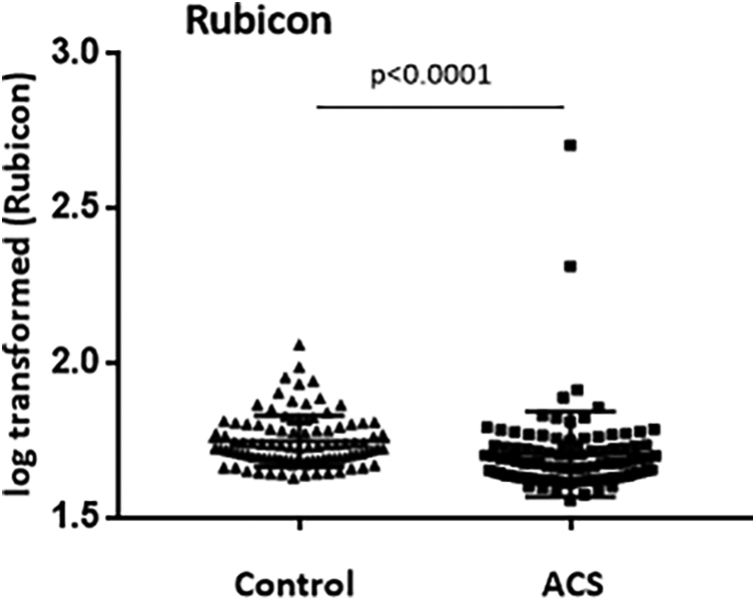
Plasma levels of Rubicon in the study population measured by enzyme-linked immunosorbent assay. The scatter plots represent the values (log transformed data) with standard deviation of control subjects (Control) and ACS patients (ACS).

The case-control study was conducted on 199 individuals. The baseline variables of the ACS and control subjects are reported in Table 1. The high percentage of hypertension, dyslipidemia or obesity, as well as the significant differences in the medical treatments found in the control group, were explained by their enrollment in the Prevention of Cardiovascular Disease department (Toulouse University Hospital Center, Toulouse, France). Plasma levels of the autophagy regulator Rubicon were quantified in the studied subjects. The mean ± SD concentration of Rubicon at baseline was statistically (*p* < 0.001) lower in ACS patients (49.92 ± 9 pg/ml) compared with control subjects (57.45 ± 12.5 pg/ml) (Figure 1). After stratification by gender, dyslipidemia, diabetes, hypertension and smoking, plasma concentrations of Rubicon were found statistically lower in diabetes subjects (Supplementary Data 1).

**Table 1:** Baseline characteristics of the study population.

		Total (*n* = 199)	ACS patients (*n* = 100)	Control subjects (*n* = 99)	*p*-value
Gender	Male	140 (70.35%)	82 (82%)	58 (58.58%)	<0.001
	Female	59 (29.64%)	18 (18%)	41 (41.41%)	
Age (years)		58.84 (8.36)	57.46 (8.84)	60.25 (7.61)	0.035
Cardiovascular risk factors					
BMI (kg/m^2^)		27.37 (4.60)	26.22 (4.13)	28.56 (4.78)	<0.001
Obesity		51 (26.15%)	15 (15.15%)	36 (37.50%)	<0.001
Dyslipidaemia		135 (69.23%)	57 (57.58%)	78 (81.25%)	<0.001
Diabetes		43 (22.05%)	19 (19.19%)	24 (25.00%)	0.3
Hypertension		129 (66.15%)	42 (42.42%)	87 (90.62%)	<0.001
Current smoking		57 (29.23%)	41 (41.41%)	16 (16.67%)	<0.001
Heredity		64 (32.82%)	29 (29.29%)	35 (36.46%)	0.3
LVEF (%)		54.94 (9.39)	51.90 (8.30)	60.09 (8.94)	<0.001
STEMI			60 (60%)		
NSTEMI			40 (40%)		
Anterior territory			36 (37.11%)		
Lower territory			36 (37.11%)		
Lateral territory			25 (25.77%)		
Blood glucose (mg/dl)		114.66 (50.35)	116.82 (41.49)	112.28 (58.72)	0.031
Triglycerides (mg/dl)		142.22 (79.81)	146.63 (87.13)	137.67 (71.65)	0.6
Total cholesterol (mg/dl)		196.71 (45.03)	195.03 (45.35)	198.45 (44.86)	0.4
LDL-cholesterol (mg/dl)^a^		118.72 (39.34)	119.56 (38.64)	117.86 (40.23)	0.8
HDL-cholesterol (mg/dl)^b^		50.23 (14.02)	47.77 (11.70)	52.77 (15.72)	0.054
Apo B (mg/dl)		99.89 (23.85)	98.28 (26.48)	100.86 (22.26)	0.4
Medical treatment at admission					
ß-blocker agents		43 (22.05%)	17 (17.17%)	26 (27.08%)	0.1
ACE inhibitors^c^		39 (20%)	15 (15.15%)	24 (25%)	0.086
Statins		73 (37.44%)	24 (24.24%)	49 (51.04%)	<0.001
Calcium channel blockers		47 (24.1%)	11 (11.11%)	36 (37.5%)	<0.001
Anti-platelet agents		43 (22.05%)	17 (17.17%)	26 (27.08%)	0.1
Anti-diabetic treatment		31 (15.9%)	10 (10.1%)	21 (21.88%)	0.025
Diuretic treatment		37 (18.97%)	2 (2.02%)	35 (36.46%)	<0.001

Data are shown as mean with standard deviation or %.

^a^ LDL, low-density lipoprotein.

^b^ HDL, high-density lipoprotei.

^c^ ACE, angiotensin-converting-enzyme.

### 3.2 Correlation between Rubicon with metabolic parameters and cardiovascular risk factors

Spearman rank correlation coefficients between Rubicon and metabolic parameters or cardiovascular risk markers were calculated. In the whole studied population, a significant positive correlation of Rubicon with lipid parameters such as total cholesterol (*r* = 0.141, *p* = 0.046), LDL (low-density lipoprotein)-cholesterol (*r* = 0.172, *p* = 0.015) and ApoB (*r* = 0.257, *p* = 0.007) was found.

### 3.3 Association of Rubicon with ACS

The results presented in Table 2 show the crude estimates and unadjusted/adjusted associations of the autophagy inhibitor Rubicon with the risk of ACS. The unadjusted logistic regression analysis revealed that Rubicon was significantly inversely associated with ACS. After adjustment for potential confounders including gender, age, obesity, dyslipidemia, hypertension, heredity, smoking and the medical treatments, the association with ACS remained statistically significant.

**Table 2:** Logistic regression analysis for Rubicon and the risk of ACS (cutoffs = quartiles).

	Non adjusted (ACS case vs. control)					Adjusted (ACS case vs. control)		
Rubicon	Point estimate	95% Wald		Pr > Khi-2	Point estimate	95% Wald		Pr > Khi-2
		Confidence limits				Confidence limits		
Q2 vs. Q1	0.237	0.097	0.576	**0**.**0015**	0.191	0.056	0.653	**0**.**0083**
Q3 vs. Q1	0.186	0.076	0.454	**0**.**0002**	0.122	0.035	0.427	**0**.**001**
Q4 vs. Q1	0.121	0.048	0.301	**<**.**0001**	0.087	0.023	0.328	**0**.**001**

Q1 = 45.6; Q2 = 50.56; Q3 = 58.67.

Adjusted for gender, age, smoking, hypertension, dyslipidemia, diabetes, obesity, heredity and medical treatments.

The bold values indicated the significantly of the *p*-value.

### 3.4 Analysis of ROC curves and ACS risk prediction for Rubicon

We then assessed whether Rubicon could increase the predictive performance of a model comprising the classical cardiovascular risk factors (CVRF) and the medical treatments. A receiver operating characteristic (ROC) curve analysis was achieved and data in Table 3 show the performance (area under the curve, AUC) of clinical model, Rubicon alone or in combination with the model. The addition of Rubicon to the model led to a significant improvement of the predictive performance from 0.868 (95% CI 0.817–0.918) to 0.896 (95% CI 0.852–0.94, *p* = 0.038). Taken together, our results highlighted an incremental value of the autophagy biomarker Rubicon to traditional cardiovascular risk factors for ACS.

**Table 3:** Receiver operating characteristic (ROC) curve analysis comparing the predictive value of the autophagy regulator Rubicon.

	AUC	95% CI	*χ* ^2^	*p-*value
Rubicon	0.6849	0.613–0.756		
CVRF model	0.868	0.817–0.918		
CVRF model + Rubicon	0.896	0.852–0.94	4.31	**0.038**

CVRF model (cardiovascular risk factors: gender, age, obesity, smoking, dyslipidemia, hypertension, diabetes, heredity and medical treatments).

AUC, area under the curve; CI, confidence interval. *χ*^2^, chi-squared (chi2) test.

The bold values indicated the significantly of the *p*-value.

## 4 Discussion

Novel reliable and sensitive biomarkers in coronary artery disease remains a major clinical need. In the present work, we explored for the first time the expression levels of plasma-circulating autophagy regulator Rubicon in a population of ACS patients compared to control subjects. Rubicon is one of the few negative regulators of autophagy. The protein inhibits autophagosome-lysosome fusion through binding to the PI3KC3 (phosphatidylinositol 3-kinase complex 3)-UVRAG (UV-irradiation-resistance-associated gene) complex (13). Levels of Rubicon increase in association with conditions of autophagy impairment such as aging (25). In our study, we could demonstrate that Rubicon levels are significantly decreased in ACS patients, which confirmed previous *in vivo* data describing autophagy activation during myocardial I/R (8). In a mouse model of myocardial I/R, ischemia stimulates autophagy through an AMPK (AMP kinase)-dependent mechanism due to the low energy and hypoxic conditions whereas reperfusion stimulates autophagy through a Beclin 1-dependent but AMPK-independent mechanism and reactive oxygen species production (8). Regarding the role of Rubicon in MI, we can only speculate based on *in vivo* studies of myocardial I/R. During the ischemic phase and the early phase of reperfusion, an increased autophagic flux and lysosomal activity is measured in the mouse myocardium, which correlates with decreased expression of the inhibitor Rubicon. However, 6 h after reperfusion the autophagic flux in the border zone decreases below basal levels coinciding with a gradually up-regulation of Rubicon (11). Therefore, on the basis of our results we hypothesized that the decreased level of circulating Rubicon in ACS patients compared to control subjects might reflect the activation of autophagy flux during ischemia and the early phase of reperfusion. Further studies are necessary to determine whether the differential expression of circulating Rubicon is a result of modulated intracellular production and/or different release mechanisms. Interestingly, a recent study showed that stressed cardiomyocytes can release large extracellular vesicles derived from autophagy which have been termed “exopheres” containing mitochondria and sarcomere proteins (26). Conceivably, we can hypothesize that autophagy mediated exocytosis can result in the release of proteins like Rubicon into the circulation. Interestingly, we also identified significant positive correlations between Rubicon and lipid parameters including total cholesterol, LDL-cholesterol and ApoB, which is in line with results showing downregulation of autophagy in heart tissues of hypercholesterolemic rats (27). Importantly, our study demonstrated for the first time that Rubicon is measured in body fluids and inversely associated with ACS independently of CVRF and medical treatments, corroborating the critical roles of Rubicon in CVD. Moreover, ROC curve analysis provided a powerful predictive model comprising Rubicon, cardiovascular risk factors and medical treatments. Indeed, the addition of Rubicon improved the predictive performance of the model alone thus bringing extra value for ACS risk prediction when included in a multi-biomarker approach. The strength and originality of the present work lies in the evaluation of two very-high-risk populations for primary and secondary prevention of cardiovascular risk. Indeed, the association of Rubicon with the risk of ACS is independent of CVRF and medical treatments, which supports the significance of circulating Rubicon as a relevant biomarker in ACS. Moreover, considering its active role in myocardial I/R, Rubicon may also represent a novel target for therapeutic interventions to reduce autosis during the reperfusion phase. Some limits in our work have to be taken into account. Though the population size had sufficient statistical power to distinguish significant differences, it would be useful to have a second independent cohort for validation. Furthermore, it would have been interesting to measure the baseline expression of Rubicon before the ACS event, nevertheless such information requires a longitudinal study of a population at high cardiovascular risk and 10-year follow-up. Moreover, our study examines circulating Rubicon levels in the acute phase, it will also be interesting to carry out a kinetics study of Rubicon expression comprising very early samples taken before 6 h, between 6 and 24 h and during a follow-up. From the perspective of our study, the discovery and the monitoring of circulating autophagy biomarkers for use in clinical practice represent a great opportunity to understand how levels of autophagy correlate with cardiac outcomes in individuals affected by CVD.

## Data Availability

The raw data supporting the conclusions of this article will be made available by the authors, without undue reservation.
