# Supplementary material for: Plasma levels of autophagy regulator Rubicon are inversely associated with acute coronary syndrome
Source: Front Cardiovasc Med. 2024 Jan 5;10:1279899. doi: 10.3389/fcvm.2023.1279899 (PMC10796531; doi:10.3389/fcvm.2023.1279899)
Supplement: Supplementary file 1 [file Table1.docx]

Supplementary Material

# Supplementary Data

| Rubicon values and cardiovascular risk factors interactions | | | | | |  |
| --- | --- | --- | --- | --- | --- | --- |
|  | n | means | SD | p value | case/control* interaction |  |
| F | 59 | 56.44 | 14.44 | 0.61 | 0.84 |  |
| M | 140 | 56.19 | 41.50 |  |  |  |
|  |  |  |  |  |  |  |
| NSTEMI | 40 | 52.93 | 25.94 | 0.84 |  |  |
| STEMI | 60 | 57.35 | 59.62 |  |  |  |
|  |  |  |  |  |  |  |
| HBP  *-* | 67 | 59.62 | 59.37 | 0.69 | 0.31 |  |
| HBP + | 132 | 54.57 | 11.654 |  |  |  |
|  |  |  |  |  |  |  |
| smoker - | 141 | 59 | 41.77 | 0.35 | 0.67 |  |
| smoker + | 58 | 49.62 | 8.04 |  |  |  |
|  |  |  |  |  |  |  |
| diabetes - | 156 | 57.99 | 39.93 | **0.036** | 0.46 |  |
| diabetes + | 43 | 50.02 | 7.25 |  |  |  |
|  |  |  |  |  |  |  |
| dyslipidemia - | 64 | 63.01 | 60.20 | 0.44 | 0.29 |  |
| dyslipidemia + | 135 | 53.07 | 11.90 |  |  |  |
|  |  |  |  |  |  |  |
| * Tests on log transformed data, SD: standard deviation | | | | |  |  |
| F, female; M, men; NSTEMI, non ST elevation myocardial infarction; STEMI, ST elevation myocardial infarction; HBP, High Blood Pressure. | | | | | | |
|  |  |  |  |  |  |  |
